# TRENDS IN NATIONWIDE RESEARCH OUTCOMES ON ELDER ABUSE AND POLICY DEVELOPMENT TO PREVENT ELDER ABUSE IN JAPAN

**DOI:** 10.1093/geroni/igae098.1848

**Published:** 2024-12-31

**Authors:** Noriko Tsukada, Asako Katsumata, Naoki Ikeda

**Affiliations:** Nihon University, Tokyo, Japan; Japan Lutheran College, Tokyo, Japan; Kamihonnmachi Sougou Houritsu Jimusyo, Osaka, Japan

## Abstract

After a brief introduction on history of activities of Japan Academy for the Prevention of Elder Abuse for the past 20 years, this study presents trends in number of elder abuse cases that were reported and substantiated as “elder abuse”, and trends in reporters and types of abuse in both institutional and domestic settings by using the nationwide data collected by the Ministry of Health, Labour, and Welfare each year since the Act on the Prevention of Elder Abuse was enacted in 2006. The number of elder abuse cases in 2022 increased 5.3% compared to that in 2021 in domestic settings, while about 17% of increase for institutional settings. And the data in 2022 showed only 7.4% of the municipalities had implemented “leadership trainings” to promote actions and measures to prevent elder abuse in institutions and agencies that provide nursing care services; accordingly, it is not surprising, without leaders, the number of elder abuse cases reported and substantiated as elder abuse increased. Moreover, although there was no amendment of the Act on the Prevention of Elder Abuse, we discuss the most recent policy amendment on “Responses to Elder Abuse and Support for Caregivers in Municipalities and Prefectures”, so-called National Manual, which was enacted in April 2023 for the first time in 5 years. This policy change required a collaboration between prefectures and municipalities to develop infrastructure to prevent elder abuse. Finally, we make recommendations for solving issues identified during the past 20 years.

